# Comparison of 2D and 4D Flow MRI in Neonates Without General Anesthesia

**DOI:** 10.1002/jmri.28303

**Published:** 2022-06-21

**Authors:** Pia Sjöberg, Erik Hedström, Katrin Fricke, Petter Frieberg, Constance G Weismann, Petru Liuba, Marcus Carlsson, Johannes Töger

**Affiliations:** ^1^ Clinical Physiology, Department of Clinical Sciences Lund Lund University, Skåne University Hospital Lund; ^2^ Diagnostic Radiology, Department of Clinical Sciences Lund Lund University, Skåne University Hospital Lund Sweden; ^3^ Pediatric Heart Center, Department of Clinical Sciences Lund Lund University, Skåne University Hospital Lund Sweden

**Keywords:** congenital heart disease, aortic coarctation, four‐dimensional flow

## Abstract

**Background:**

Neonates with critical congenital heart disease require early intervention. Four‐dimensional (4D) flow may facilitate surgical planning and improve outcome, but accuracy and precision in neonates are unknown.

**Purpose:**

To 1) validate two‐dimensional (2D) and 4D flow MRI in a phantom and investigate the effect of spatial and temporal resolution; 2) investigate accuracy and precision of 4D flow and internal consistency of 2D and 4D flow in neonates; and 3) compare scan time of 4D flow to multiple 2D flows.

**Study Type:**

Phantom and prospective patients.

**Population:**

A total of 17 neonates with surgically corrected aortic coarctation (age 18 days [IQR 11–20]) and a three‐dimensional printed neonatal aorta phantom.

**Field Strength/Sequence:**

1.5T, 2D flow and 4D flow.

**Assessment:**

In the phantom, 2D and 4D flow volumes (ascending and descending aorta, and aortic arch vessels) with different resolutions were compared to high‐resolution reference 2D flow. In neonates, 4D flow was compared to 2D flow volumes at each vessel. Internal consistency was computed as the flow volume in the ascending aorta minus the sum of flow volumes in the aortic arch vessels and descending aorta, divided by ascending aortic flow.

**Statistical tests:**

Bland–Altman plots, Pearson correlation coefficient (*r*), and Student's *t*‐tests.

**Results:**

In the phantom, 2D flow differed by 0.01 ± 0.02 liter/min with 1.5 mm spatial resolution and −0.01 ± 0.02 liter/min with 0.8 mm resolution; 4D flow differed by −0.05 ± 0.02 liter/min with 2.4 mm spatial and 42 msec temporal resolution, −0.01 ± 0.02 liter/min with 1.5 mm, 42 msec resolution and −0.01 ± 0.02 liter/min with 1.5 mm, 21 msec resolution. In patients, 4D flow and 2D flow differed by −0.06 ± 0.08 liter/min. Internal consistency in patients was −11% ± 17% for 2D flow and 5% ± 13% for 4D flow. Scan time was 17.1 minutes [IQR 15.5–18.5] for 2D flow and 6.2 minutes [IQR 5.3–6.9] for 4D flow, *P* < 0.0001.

**Data Conclusion:**

Neonatal 4D flow MRI is time efficient and can be acquired with good internal consistency without contrast agents or general anesthesia, thus potentially expanding 4D flow use to the youngest and smallest patients.

**Evidence Level:**

1

**Technical Efficacy:**

Stage 2

Children with critical congenital heart disease (CHD) may require surgery or endovascular interventions during the first weeks of life to survive.^1^, ^2^ Accurate and precise blood flow measurements are important for intervention planning.^3^, ^4^, ^5^ Whereas echocardiography is the standard modality for the assessment of anatomy and cardiac function in neonates,^5^, ^6^ CT can offer good anatomical assessment in cases where visualization is not adequate with echocardiography. However, CT has the disadvantage of ionizing radiation. MRI, on the other hand, can provide anatomy without the use of ionizing radiation, and at the same time assess function and blood flow. However, MRI is hampered by long examination times and therefore general anesthesia may be needed to avoid body movements. General anesthesia adds risk to the examination,^7^ may change the physiology and has been shown to result in depressed ejection fraction in patients with CHD.^8^ Furthermore, general anesthesia adds costs since it requires skilled anesthetists and nurses familiar with the MRI environment, as well as facilities for recovery afterwards.

Standard through‐plane phase‐contrast MRI (2D flow) acquires data in a single plane and therefore multiple acquisitions are needed to measure flow in all vessels of interest. This means that scan duration can be extensive in patients with complex CHD and highly trained scanner operators are required.^9^ Recent developments in three‐dimensional (3D) time‐resolved three‐directional phas‐contrast MRI (four‐dimensional [4D] flow) have enabled shorter scan duration as flows in all vessels of interest are acquired simultaneously and requires minimal slice planning compared to two‐dimensional (2D) flow.^10^, ^11^ This shorter scan duration increases the chance of successful study when using feed‐and‐sleep,^12^ instead of sedation or general anesthesia. However, most studies on accuracy and precision of 4D flow have been performed in adults or adolescents,^13^, ^14^, ^15^, ^16^, ^17^, ^18^ rather than in neonates with small vessels. Furthermore, 4D flow visualization improves with use of contrast agent,^19^ which carries the potential risks of gadolinium deposition and nephrogenic systemic fibrosis.^20^ There is limited published data on use of contrast agents in neonates, but it is recommended to avoid contrast agents in neonates if possible.^20^

Therefore, the purpose of this study was to investigate the accuracy, precision and internal consistency of 4D flow measurements in neonates without general anesthesia or contrast agent with the free breathing feed‐and‐sleep approach. Specifically, the aims were to 1) validate 2D‐ and 4D flow measurements using a controlled, pulsatile flow phantom and to investigate the effect of spatial and temporal resolution, 2) compare 4D flow to reference 2D flow measurements and assess the internal consistency of 2D and 4D flow in neonates after surgery for aortic coarctation, and 3) compare acquisition duration for multiple 2D flow scan planes to a single 4D flow acquisition.

## Materials and Methods

### MRI Scans

MRI acquisitions in the phantom and in patients were performed on a 1.5 T MRI MAGNETOM Aera (Siemens Healthcare, Erlangen, Germany).

#### FLOW PHANTOM

**FIGURE 1:**
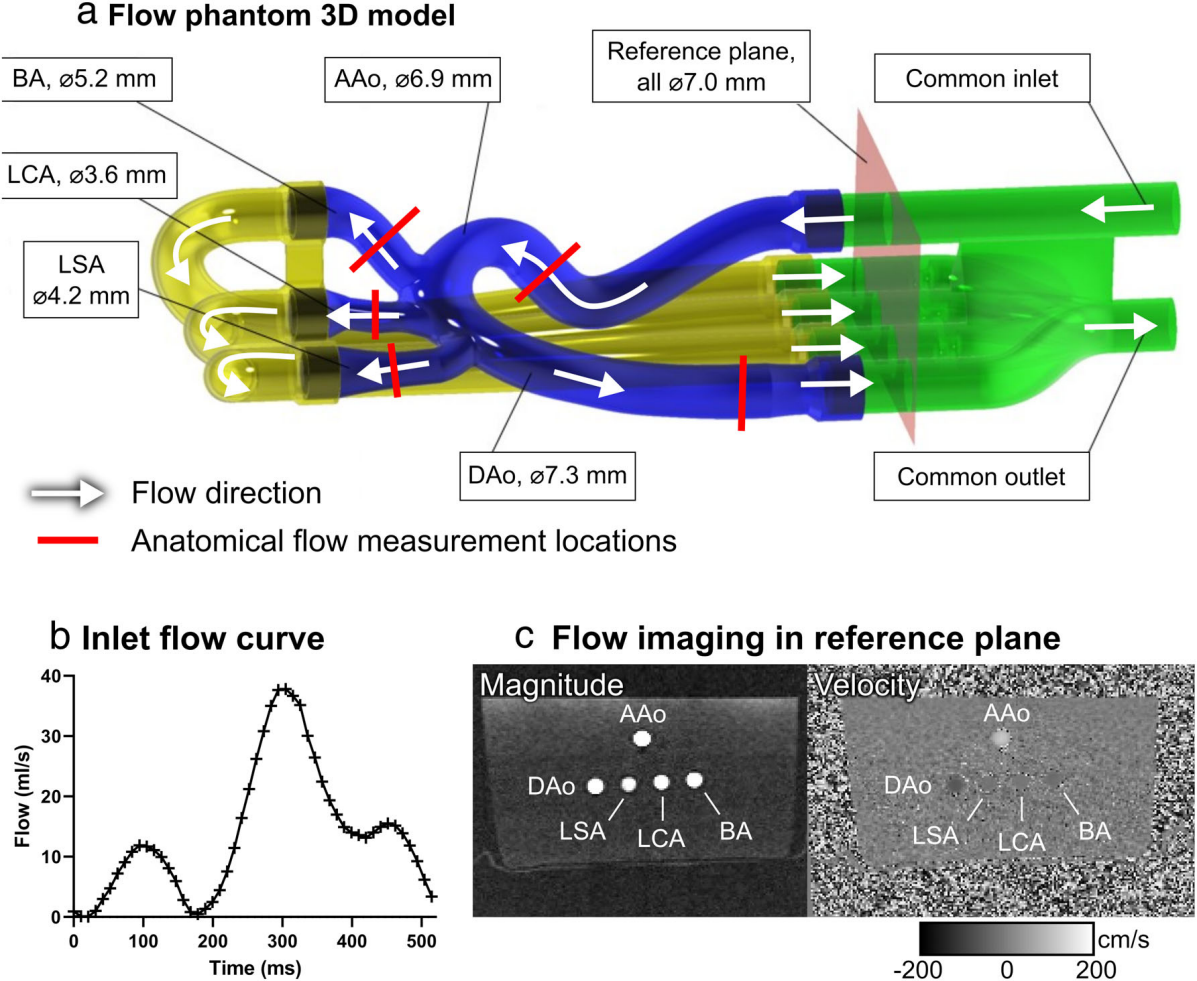
Flow phantom model. The pulsatile pump, set to 120 beats/minute and a net flow of 0.75 liters/minute, was connected to the “common inlet,” which was connected to the ascending aorta (Aao). The three outlets on the aortic arch correspond to the aortic arch vessels in patients: (1) the brachiocephalic artery (BA), (2) the left carotid artery (LCA), and (3) left subclavian artery (LSA). The three aortic arch vessels and the descending aorta (DAo) then merge into a “common outlet” that was returned to the pump. The common inlet (ascending aorta), the descending aorta and the three aortic arch vessels at the aortic arch were arranged in parallel so that a single 2D flow measurement with high spatial resolution could be acquired in a reference plane to measure flow in all branches simultaneously. The total flow rate was verified using timer and beaker at the common outlet. (a) Flow phantom 3D model; (b) inlet flow curve; (c) flow imaging in reference plane.

A 3D‐printed phantom model (Raise3D Pro 2, Irvine, CA, USA) of a neonatal aorta based on MR images acquired in one of the patients in the study was constructed in Creo Parametric (PTC, Boston, MA, USA) and used to assess measurement accuracy in a controlled setting. The phantom model was printed in a stiff (noncompliant) plastic material and consisted of ascending and descending aorta, brachiocephalic artery, left common carotid artery, and left subclavian artery. The phantom was connected to a pump generating pulsating flow at a frequency of 120 beats per minute (Fig. 1a). Net flow through the common outlet (equal to the inlet, i.e. ascending aorta flow) was measured using timer and beaker before and after MRI flow scans. Gadolinium contrast agent (Dotarem, Guerbet, Villepinte, France) was added to the flowing water, resulting in T_1_ ≈ 1700 msec, close to that of blood at 1.5 T, and to the water surrounding the tubes resulting in T_1_ ≈ 1050 msec, close to that of skeletal muscle or myocardium.

Flow was measured at the anatomical vessel locations using the clinical 2D flow sequence, that is, the locations in the phantom corresponding to where flow would be measured in vivo (see Fig. 1a). To obtain reliable reference flow measurements of the aortic arch vessels and the descending aorta, the tubes were organized such that after leaving the aorta they turned around and passed through a common “reference plane” where all tubes were straight, oriented in the feet‐head direction and of the same diameter (7 mm, see Fig. 1). The larger diameter enables high accuracy flow measurements due to a higher number of pixels per tube diameter^21^ compared to the anatomical locations. The purpose of the straight course of the tubes was also to avoid secondary flow patterns that could degrade flow measurements and to reduce error due to angulation.^21^ Since the flow in a stiff tube is the same regardless of where along the tube it is measured, a flow measurement at the reference plane can be used as an accurate reference for flow measurements at the anatomical locations.

Sequence parameters for phase‐contrast flow imaging are shown in Table 1. Flow was measured at the anatomical locations using the 2D flow sequence used in the patients (spatial resolution 1.5 mm × 1.5 mm × 5 mm) and also with a sequence with higher spatial resolution (0.8 mm × 0.8 mm × 5 mm). The higher spatial resolution was used for reference plane measurements.

**TABLE 1:** MRI Sequence Parameters

Parameters	Black‐Blood T1‐Weighted	2D Flow		4D Flow			
		Patient Protocol	Improved Spatial Resolution	Patient Protocol	Improved Spatial Resolution	Improved Temporal Resolution	Improved Spatial and Temporal Resolution
Used in:	Patients	Patients and phantom	Phantom	Patients and phantom	Phantom	Phantom	Phantom
Field of view (read × phase × slice, mm)	326 × 245 × 40	320 × 259 × 5	320 × 258 × 5	380 × 285 × 60	240 × 180 × 66	380 × 285 × 60	240 × 180 × 66
Acquired spatial resolution (read × phase × slice, mm)	0.85 × 0.85 × 0.5	1.5 × 1.5 × 5	0.8 × 0.8 × 5	2.4 × 3.6 × 1.5	1.5 × 1.5 × 1.5	2.4 × 3.6 × 1.5	1.5 × 1.5 × 1.5
Reconstructed spatial resolution (read × phase × slice, mm)	0.85 × 0.85 × 0.5	1.5 × 1.5 × 5	0.8 × 0.8 × 5	2.4 × 2.4 × 1	1.5 × 1.5 × 1.5	2.4 × 2.4 × 1	1.5 × 1.5 × 1.5
Temporal resolution, acquired (msec/frames)	n/a	9.8/44 ± 4 frames per heart beat	12.4/40 frames per heart beat	42/11 ± 1 frames per heart beat	42/12 frames per heart beat	21/24 frames per heart beat	42/24 frames per heart beat
Temporal resolution, reconstructed	n/a	35 frames per heart beat	50 frames per heart beat	20 frames per heart beat	20 frames per heart beat	25 frames per heart beat	25 frames per heart beat
Temporal segmentation factor	n/a	1	1	2	2	1	1
TR/TE/flip (msec/msec/°)	650/20/120	4.9/2.7/20	6.2/3.6/20	5.2/2.5/7	5.2/2.5/7	5.2/2.5/7	5.3/2.5/7
Averages	1.8	1	1	1	1	1	1
Bandwidth per pixel (Hz)	870	455	445	455	455	455	455
VENC, cm/sec	n/a	200	200	150	150	150	150
Acceleration	GRAPPA, R = 2	GRAPPA, R = 2	GRAPPA, R = 2	GRAPPA, R = 2	GRAPPA, R = 2	GRAPPA, R = 2	GRAPPA, R = 2
Cardiac cycle gating	Prospective, ECG	Retrospective, patients: ECG, phantom: PPU	Retrospective, PPU	Retrospective, patients: ECG, phantom: PPU	Retrospective, PPU	Retrospective, PPU	Retrospective, PPU
Respiratory gating	No	No	n/a	No	n/a	n/a	

TE = echo time, TR = repetition time, flip = flip angle, VENC = velocity encoding parameter, n/a = not applicable.

4D flow data were acquired using a prototype sequence (i.e. a development version for research purposes, not marketed by the manufacturer as a product) with acquired spatial resolution of 2.4 mm × 3.6 mm × 1.5 mm (as subsequently used in patients) (Table 1). In addition, 4D flow data were acquired with higher spatial and temporal resolutions. Sequence details for 2D and 4D flow and for T1‐weighted black blood acquisitions (used for anatomical visualization and creating the phantom) are provided in Table 1. The 4D flow sequence included compensation for concomitant gradients.

#### PATIENTS

**FIGURE 2:**
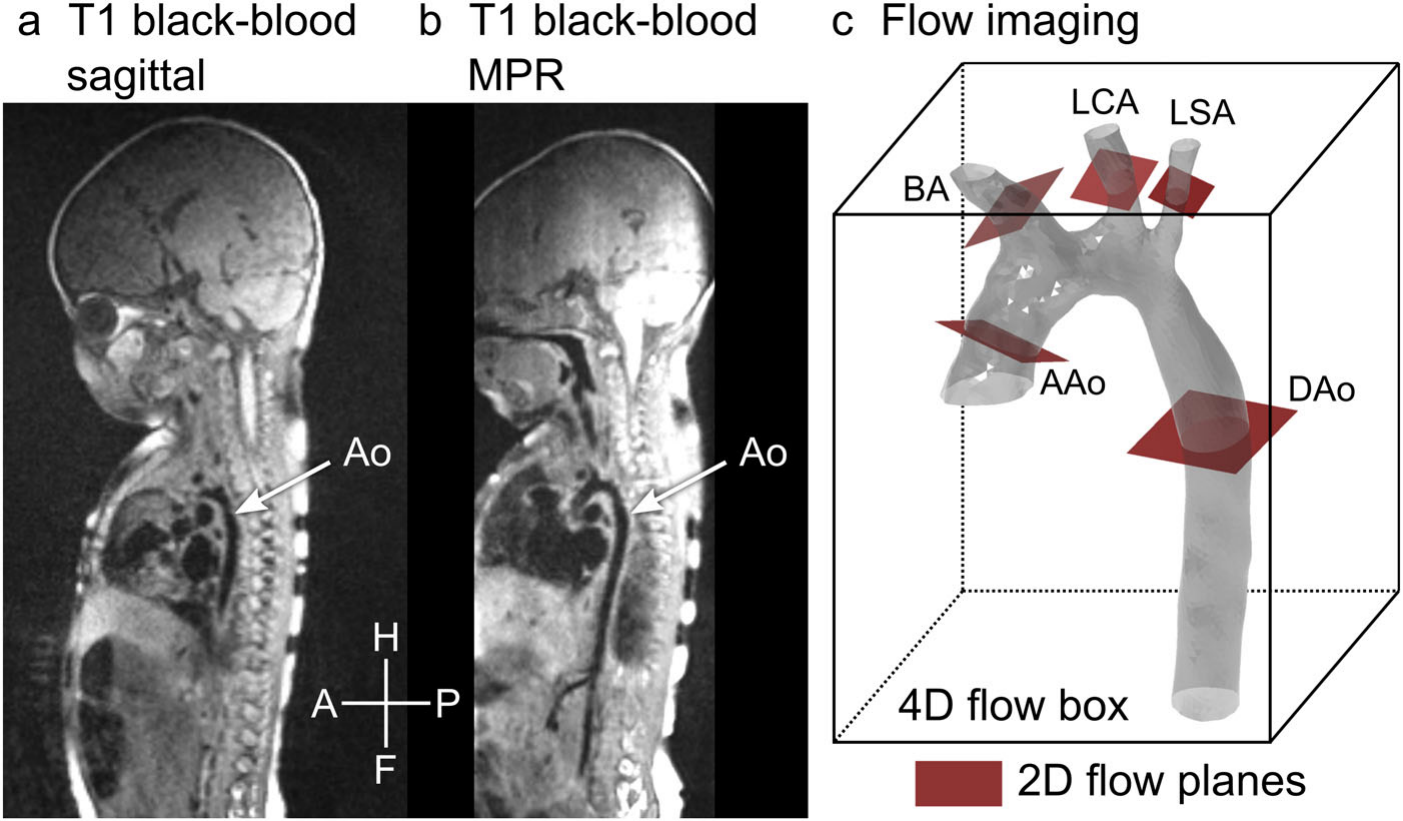
Overview and scan planning. Panel (a) shows a T1‐weighted black‐blood image taken in the sagittal view. A stack of such images was used to find locations for 2D flow measurement. The aorta is partially visible. Panel (b) shows the same image stack displayed at an angle using multiplanar reformatting (MPR), with a better view of the aorta. Panel (c) shows schematically how flow imaging was performed. The gray mesh shows a 3D model of the aorta, the box represents a 4D flow measurement covering the whole aorta and the red squares show locations where 2D flow was acquired. H/F = head/feet, A/P = anterior/posterior. AAo = ascending aorta, Dao = descending aorta, BA = brachiocephalic artery, LCA = left carotid artery, LSA = left subclavian artery, MPR = multiplanar reconstruction.

Seventeen neonates, age 18 days [IQR 11–20], with surgically corrected aortic coarctation and without associated major CHD were included 5 days [IQR 4–8] after surgery between November 2018 and October 2020. The Ethical Review Board approved the study and parents gave written informed consent prior to MRI. The principles of the Helsinki declaration were followed. The MRI scan was performed as a research scan using feed‐and‐sleep^12^ supported by chloral hydrate if needed. The neonates were fasting prior to scan preparations. Fasting time was kept to the normal feeding interval for the neonate. Just before the examination, neonates were fed, and diapers changed. If the neonate did not settle, chloral hydrate (25 mg/kg, APL, Sweden) was administered rectally. After applying hearing protection, the neonates were wrapped in blankets and put to rest in a vacuum immobilizer by their parents. A nurse stayed with the neonate during the examination to provide comfort if needed. A higher resolution T1‐weighted black‐blood 3D sequence was used to depict vessel anatomy. Flow volumes were measured with 2D flow at the ascending aorta, descending aorta, brachiocephalic artery, left common carotid artery, and left subclavian artery, and with the 4D flow sequence covering the aorta (Fig. 2). Respiratory gating for 2D flow was omitted since adding it would prolong scan time. Sequence parameters are shown in Table 1. No contrast agent was used. A transthoracic echocardiogram (echo) was conducted at the same day as the MRI prior or straight after the MRI scan. The echo was performed by one of two experienced echocardiographers (K.W. with 20 years of experience and Z.A. with 8 years of experience) following a detailed echo protocol. For all examinations, a Philips Epiq 7 echo machine was used (Philips Medical System, Andover, MA, USA).

### Flow Analysis

4D flow was evaluated using 2D flow as reference. Flow images were analyzed using Segment (Medviso AB, Lund, Sweden)^22^ with an in‐house developed research module for 4D flow where the delineations performed in the 2D dataset were transferred to the 4D flow dataset to ensure that 2D and 4D flow parameters were measured at the same position. Furthermore, 4D flow was analyzed in CAAS MR Solutions 5.1.1 (Pie Medical Imaging, Maastricht, the Netherlands) with flow planes retrospectively placed, to assess intersoftware analysis and to assess the possible impact of differences in plane position and region of interest (ROI) size in patients.

Phase background was corrected by subtracting a first‐order fit to stationary tissue in 4D flow and 2D flow data.^23^, ^24^ Phase unwrapping was not needed.

#### PHANTOM

All phantom analyses were performed using the Segment software. Net flow volumes at the in the tubes representing ascending and descending aorta, the brachiocephalic artery, left carotid artery, and left subclavian artery were measured were measured with 2D flow with high spatial resolution at the reference plane. These values were then used as reference for the 2D flow and 4D flow measurements at the anatomical positions. Two blinded readers (P.S. with 9 and J.T. with 13 years of experience in cardiac MRI) performed 2D and 4D flow measurements for interobserver variability. Internal consistency in the flow measurements was measured as the difference between the inflow (ascending aorta) and outflow (sum of aortic arch vessels and descending aorta). In addition, the internal consistency was expressed as percentage, for example, the difference divided by the flow in the ascending aorta.

#### PATIENTS

Net flow volumes by 4D flow were compared to 2D flow volumes in the ascending and descending aorta, the brachiocephalic artery, left carotid artery, and left subclavian artery. Internal consistency in 2D flow and 4D flow was computed as described above. Maximum flow rate in the ascending aorta by 4D flow was compared to 2D flow.

#### INTEROBSERVER AND INTERSOFTWARE ANALYSIS IN PATIENTS

Two blinded readers (P.S. with 9 and E.H. with 21 years of experience in cardiac MRI) performed 2D and 4D flow measurements for interobserver variability. One observer (P.S. with 9 years of experience in cardiac MRI and with the software Segment and 1 year experience with the software CAAS MR Solutions) assessed intersoftware variability for 4D flow volumes between Segment (Medviso AB) and CAAS MR solutions 5.1.1 (Pie Medical Imaging, Maastricht, the Netherlands).

#### SCAN ACQUISITION TIME FOR 4D vs. 2D FLOW

Acquisition time for 2D flow was computed as the sum of all 2D flow acquisitions and compared to the acquisition time for the 4D flow sequence. Time to acquire localizers, bSSFP anatomical images, 3D T1w sequence, and planning was added to 2D flow acquisition time to compute total scan time for 2D flow. Planning time for 2D flow was not measured as part of the study, however, based on experience at our center mean planning time per 2D flow is approximately 1 minute in this patient group. Time to acquire localizers was added to the 4D acquisition time to compute total scan time for 4D flow.

#### PEAK VELOCITY IN THE ISTHMUS REGION

Peak velocity in the isthmus region was analyzed with 4D flow using CAAS MR Solutions and compared to peak velocity measured by continuous wave doppler.

### Statistical Analysis

Statistical analysis was performed using GraphPad Prism (GraphPad Software Inc., La Jolla, CA, USA) and Mangold, Pascal (2018) ICC calculation software based on Wirtz and Caspar 2002 (www.mangold-international.com). Continuous variables are presented as mean (standard deviation [SD]) for normally distributed data or median (interquartile range [IQR]) if not normally distributed. D'Agostino and Pearson normality test was used to determine the normality. Categorical variables are presented as absolute numbers and percentages. Comparisons between readers and methods, respectively, were performed using Bland–Altman plots, interclass correlation (ICC), Pearson correlation coefficient (*r*) or Student's *t*‐test. *P* values < 0.05 were considered to show statistically significant differences.

## Results

### Phantom Validation

Timer and beaker measurement showed a net flow of 0.82 liter/min both before and after flow scans. There was no difference between 2D flow volumes (0.82 liter/min) vs. timer and beaker (0.82 liter/min) in the tube representing the ascending aorta (common inlet) at the reference plane. The sum of flow volumes measured in the other tubes at reference plane was 0.07 liter/min higher than in the ascending aorta tube.

Tube diameters at the anatomical locations were 6.9 mm in the ascending aorta, 7.3 mm in the descending aorta, 5.2 mm and in the brachiocephalic artery, 3.6 mm in the left carotid artery and 4.2 mm in the left subclavian artery (Fig. 1a). There was a significant and strong correlation between the phantom flows measured with 2D flow (*r* = 1.00; ICC 1.0) and 4D flow (*r* = 1.0; ICC 1.00) by two observers. The mean difference between observers was 0.00 ± 0.02 liter/min for 2D flow and 0.00 ± 0.01 liter/min for 4D flow.

The 2D flow and 4D flow volumes at anatomical planes in the 3D phantom using different spatial and temporal resolutions were compared to the 2D flow volume measurements at reference plane, results are presented in Table 2. There was no statistically significant difference between either 2D or 4D flow and the 2D reference flow.

**TABLE 2:** Results From Phantom study

Difference in 2D and 4D Flow at Anatomical Planes vs 2D Flow at Reference Plane (liter/min)						
Flow Plane	Ascending Aorta	Descending Aorta	Brachiocephalic Artery	Left Carotid Artery	Left Subclavian Artery	Mean
2D 1.5 mm	0.04	0.03	0.00	−0.02	0.00	0.01
2D 0.8 mm	0.02	0.00	−0.02	−0.02	−0.01	−0.01
4D 2.4 mm 42 msec	−0.06	−0.05	−0.06	−0.04	−0.02	−0.05
4D 1.5 mm 42 msec	0.03	−0.01	−0.03	−0.02	−0.01	−0.01
4D 1.5 mm 21 msec	0.00	−0.02	−0.04	−0.01	0.00	−0.01

The internal consistency for 2D flow (difference between flow in the ascending aorta and sum of the flow in the aortic arch vessels and descending aorta) was −0.03 liter/min or 3% at 1.5 mm in‐plane resolution and −0.01 liter/min or 1% at 0.8 mm in‐plane resolution. For 4D flow, internal consistency was −0.04 liter/min or −6% at 2.4 mm isotropic resolution and 42 msec temporal resolution (the resolution used in patients), −0.03 liter/min or 4% with 1.5 mm and 42 ms and −0.00 liter/min or 0% with 1.5 mm and 21 msec.

Peak velocity in the ascending aorta measured with 2D and 4D flow at different resolutions are reported in Fig. 6.

### Patient Data

Patient characteristics are shown in Table 3. All 17 neonates slept through the examination and all flow acquisitions were successful in all neonates. Sixteen patients received chloral hydrate as adjunct to feed‐and‐sleep.

**TABLE 3:** Patient Characteristics

Neonates with Aortic Coarctation, *n* = 17	
Median [IQR] or mean ± SD	
Sex	10 girls/7 boys
Body surface area (BSA) (m^2^)	0.21 ± 0.02
Heart rate (bpm)	140 ± 13
Age at surgery (days)	8 [IQR 5–16]
Age at MRI (days)	18 [IQR 11–20]
Time between surgery and MRI (days)	5 [IQR 4–8]
Operation	Extended end‐to‐end anastomosis (*n* = 11) Arch reconstruction with homograft patch (*n* = 6)

BSA = body surface area.

**FIGURE 3:**
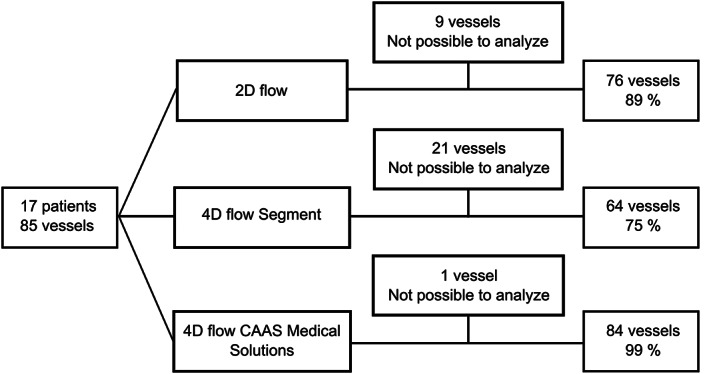
Flow diagram. Number of vessels with successful flow measurements with 2D flow and 4D flow using either segment or CAAS MR solutions.

Flow data were acquired with both 2D and 4D sequences in all patients. Exclusion criteria were that it was not possible to ensure the anatomical position of the flow measurement assisted by the anatomical images due to slight movement by the neonate or that the plane was not perpendicular to the flow. Nine vessels were excluded from 2D flow analysis (Fig. 3). To analyze 4D flow in segment the ROI made in the 2D flow dataset is transferred to the 4D flow dataset. Thus, the 2D flow plane must be spatially aligned with the 4D flow dataset. If the patient had moved during the examination, it was not always possible to co‐register 2D and 4D flow, which led to exclusion of additional 12 vessels. In CAAS MR solutions, the ROIs are drawn in the 4D flow dataset directly, based on 3D visualization of the 4D flow data. Thus, almost all vessels could be located successfully, except for the left subclavian artery which could not be located in one patient. The decision to exclude a segment from analysis was made by the analyzing observer.

**FIGURE 4:**
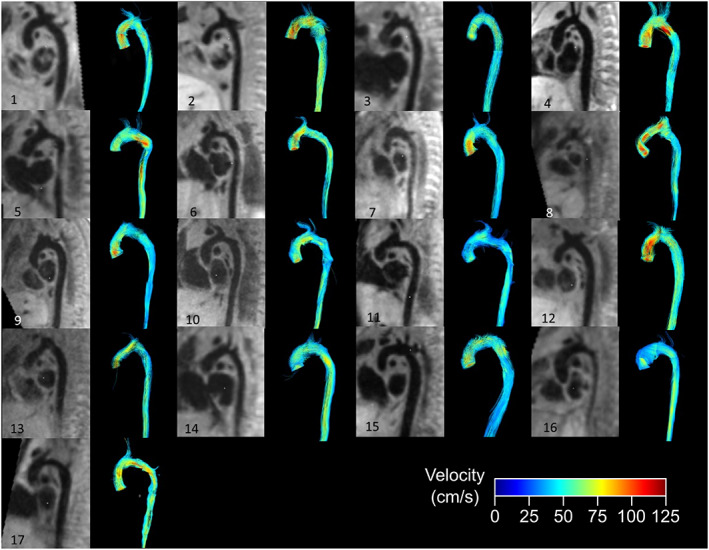
Visualized results. Anatomy of the aortic arch after operation for aortic coarctation in 17 neonates shown with MPR from a high‐resolution T1‐weighted black‐blood 3D sequence using segment and streamlines from 4D flow using CAAS MR solutions.

Vessel diameters and ROI areas were 6.9 ± 1.1 mm and 33.9 ± 17 mm^2^ in the ascending aorta, 5.1 ± 1.2 mm and 22.0 ± 12.5 mm^2^ in the descending aorta, 3.4 ± 0.6 mm and 8.2 ± 4.2 mm^2^ in the brachiocephalic artery, 2.5 ± 0.4 mm and 4.6 ± 2.3 mm^2^ in the left carotid artery and 2.3 ± 0.5 mm, and 4.0 ± 2.2 mm^2^ in the left subclavian artery. Visualizations of all patients' aortic arch anatomy derived from the T1‐weighted sequence using Segment and streamlines from 4D flow using CAAS MR Solutions are shown in Fig. 4.

**FIGURE 5:**
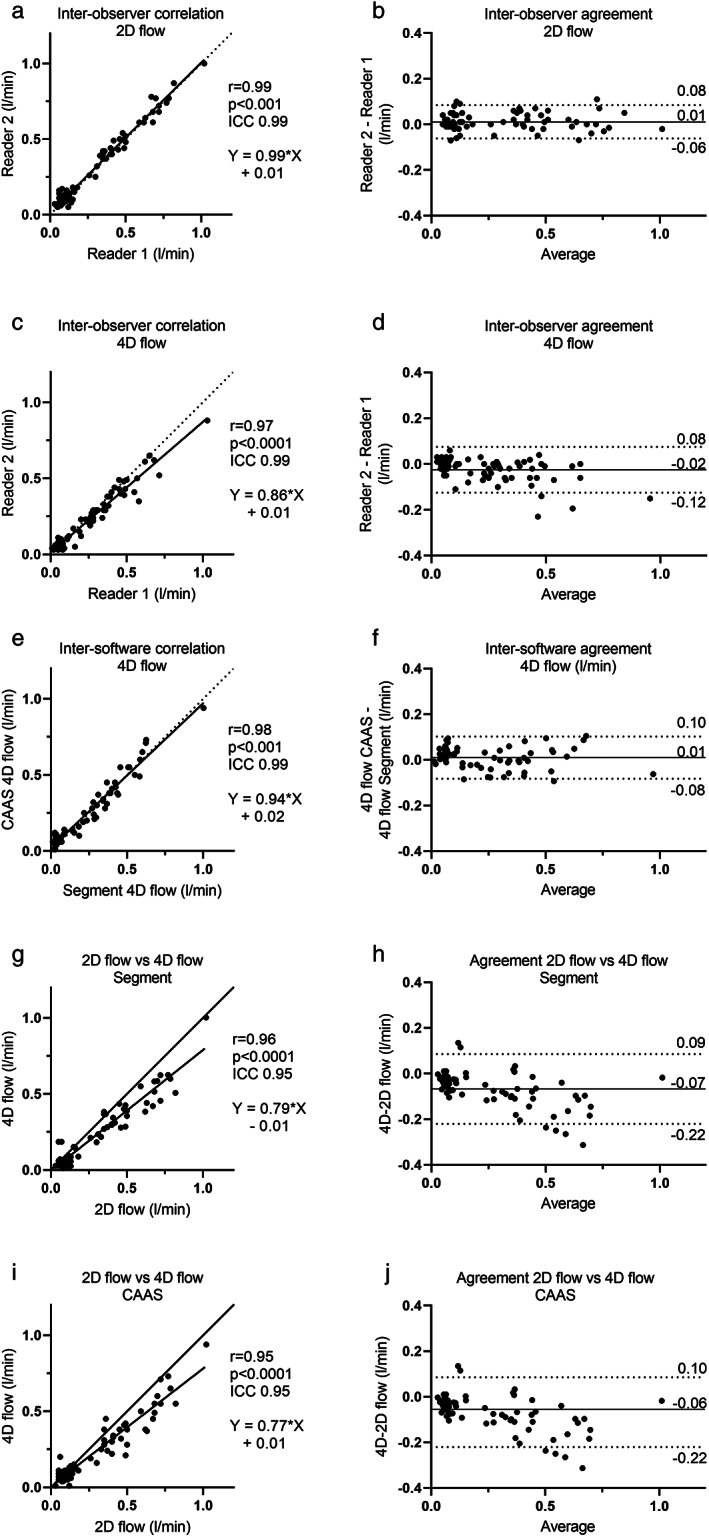
Quantitative flow results. (a) Scatter plot of 2D flow measurements showing agreement between readers. (b) Bland–Altman plot of 2D flow measurements showing interobserver agreement with a bias of 0.01 ± 0.04 liter/min. (c) Scatter plot of 4D flow measurements showing agreement between readers. (d) Bland–Altman plot of 4D flow measurements showing interobserver agreement with a bias of −0.02 ± 0.05 liter/min. Panel (e) scatter plot of 4D flow measurements showing correlation between different software. (f) Bland–Altman plot of 4D flow measurements showing intersoftware agreement with a bias of 0.01 ± 0.05 liter/min. (g,i) Scatter plot showing correlation between 2D flow and 4D flow using CAAS MR solutions (g) and segment. (h,j) Bland–Altman plot showing that 4D flow volumes analyzed either with segment (h) (bias of −0.07 ± 0.08) or CAAS Medical Solutions (j) (bias of −0.06 ± 0.08) was lower than 2D flow volumes.

**FIGURE 6:**
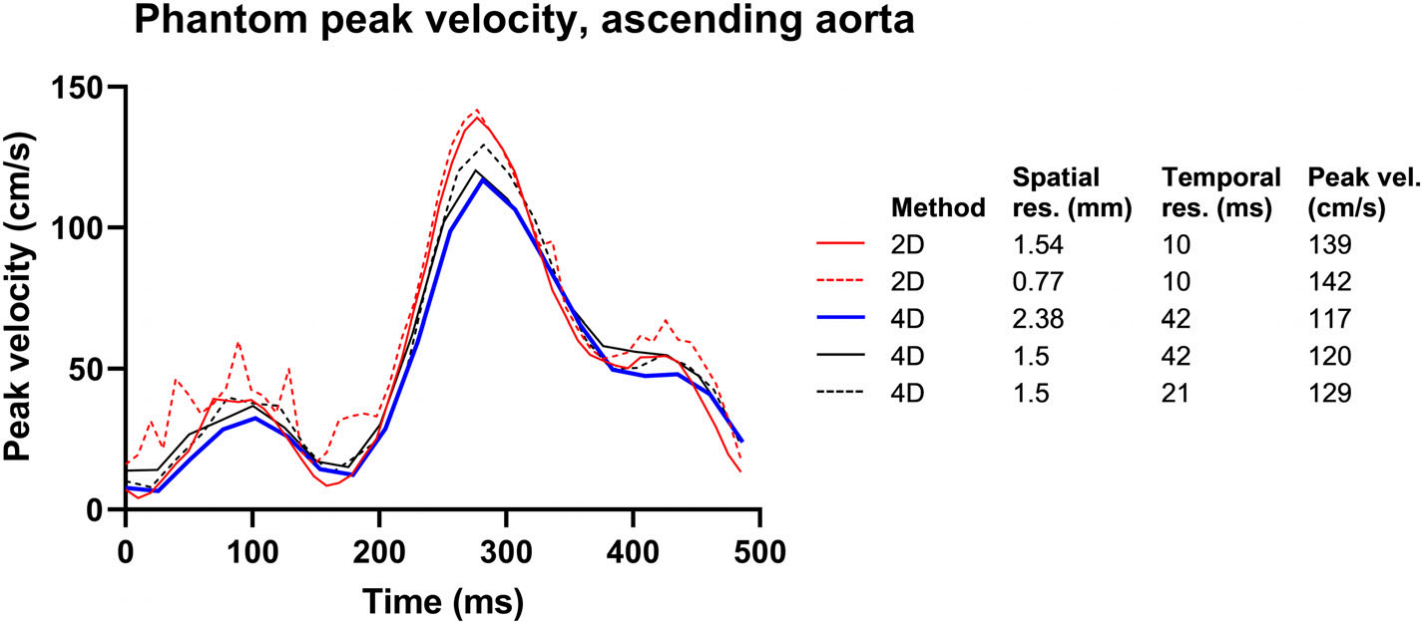
Phantom results of peak velocities. Curve showing the peak velocity during a phantom cardiac cycle with different spatial and temporal resolutions. 4D flow underestimated the peak velocity. Increased spatial resolution gave a small increase in peak velocity in both 2D and 4D flow. Adding increased temporal resolution in 4D flow increased the peak velocity.

The interobserver variability for 2D flow was *r* = 0.99, ICC 0.99, with a bias of 0.01 ± 0.04 liter/min (Fig. 5a,b) and for 4D flow *r* = 0.97, ICC 0.99, with a bias of −0.02 ± 0.05 liter/min (Fig. 5c,d). The intersoftware correlation coefficient was *r* = 0.98, ICC 0.99, with a bias of 0.01 ± 0.05 liter/min (Fig. 5e,f). The correlation coefficient for 4D flow volumes compared with 2D flow volumes was *r* = 0.96, ICC 0.95, with a bias of −0.07 ± 0.08 using Segment software (Fig. 5g,h) and *r* = 0.95, ICC 0.95, with a bias of −0.06 ± 0.08 using CAAS MR Solutions (Fig. 5i,j). The difference between 2D flow and 4D flow using CAAS MR Solutions or Segment was statistically significant (*P* < 0.0001) (Fig. 6).

Internal consistency assessed by correlating flow in the ascending aorta and the sum of flow volumes in the aortic arch vessels and descending aorta, with 2D flow resulted in a correlation coefficient of *r* = 0.72, ICC 0.81, and a bias of −0.07 ± 0.11 liter/min or −11% ± 17% (*n* = 12). When assessed with 4D flow and analyzed with Segment the correlation coefficient was *r* = 0.97, ICC 0.89, with a bias of 0.01 ± 0.07 liter/min or 1 ± 16% (*n* = 9) and when analyzed with CAAS MR Solutions the correlation coefficient was *r* = 0.91, ICC 0.94, with a bias of 0.03 ± 0.08 liter/min or 5% ± 13% (*n* = 16).

For maximum flow rate, 4D flow measurements in the ascending aorta were lower than 2D flow (41.8 ± 11.8 mL/sec), both when analyzed with Segment (30.3 ± 10.0 mL/sec, *P* < 0.0001) and CAAS (29.2 ± 8.5 mL/sec, *P* < 0.0001) with a difference of −11.3 ± 7.7 mL/sec with Segment and −12.6 ± 7.5 mL/sec with CAAS.

Median time to acquire overview anatomical images, planning and acquiring the 4D flow acquisition was 6.2 [IQR 5.3–6.9] minutes. The corresponding time for 2D flow was in total 17.1 [IQR 15.5–18.5] minutes, *P* < 0.0001. Median acquisition time for the multiple 2D flow acquisitions (excluding planning time) was 4.8 minutes [IQR 3.9–5.4], and for 4D flow 5.1 minutes [IQR 4.2–5.5], *P* = 0.43.

Mean postoperative peak velocity across the aortic isthmus was 1.2 ± 0.3 m/sec by 4D flow (CAAS MR Solutions) and 1.4 ± 0.3 m/sec by continuous wave Doppler (p = 0.054). The correlation coefficient *r* was 0.52, ICC 0.64, with a bias of −0.1 ± 0.3 m/sec.

## Discussion

This study demonstrated the feasibility of acquiring 4D flow data in neonates without the need for contrast agents or general anesthesia. Phantom validation showed a small underestimation of flow volumes by 4D flow and a slight overestimation by 2D flow using the temporal and spatial resolutions used in patients. Increasing spatial resolution improved the accuracy for both 2D and 4D flow. Increasing 4D flow temporal resolution did not improve the accuracy of flow volumes; however, it improved internal consistency and reduced the underestimation of peak velocity. Patient 4D flow volumes were lower than 2D flow volumes, but with high correlation between 2D and 4D flow and good internal consistency. When taking planning into account, 4D flow is faster than multiple 2D flow slices. In summary, 4D flow can be extended to the youngest and smallest patients, adding to previous studies in adults and adolescents showing good agreement between 4D and 2D flow.^16^, ^18^, ^25^, ^26^

### Accuracy and Precision With 2D Flow

2D flow is accurate in small vessels^27^ and was therefore used as the reference standard in this study and the accuracy for 2D flow volumes in the ascending aorta was good compared to timer and beaker in the phantom. Nevertheless, 2D flow in the phantom experiment showed an overestimation of flow volumes in the aortic arch vessels and descending aorta of 8%, compared to the ascending aorta using 2D flow, most likely explained by the limited spatial resolution^28^ in relation to the vessel diameter, an effect that is more pronounced in smaller vessels, for example, the aortic arch vessels in neonates. Acquisition with increased in‐plane spatial resolution in the phantom model improved accuracy of 2D flow in the larger vessels, together with improved internal consistency. The lack of improvement of accuracy in the smaller vessels may in part be explained by the low flow volumes and that the difference in measured flow is in the range of interobserver variability. It may, however, also in part be because of the unchanged slice thickness of 5 mm, which has been shown to lead to overestimation of flow volumes when the flow direction is not perpendicular to the imaging slice.^28^

Interobserver variability of 2D flow in both phantom and patients was low and in line with published data in adults.^16^, ^29^ However, in relation to the small vessels and low flow volumes, this small variability in absolute numbers is more important in relative terms and can be of clinical importance. 2D flow might overestimate flow volumes by up to 20% when ROI size is larger than the vessel and even more when the imaging plane is not perpendicular to the flow,^27^, ^28^ and a ROI that is too small leads to a corresponding underestimation in flow (i.e. a 10% too small ROI area will give a 10% underestimation of flow).

### Accuracy and Precision with 4D Flow

Flow volumes measured with 4D flow in patients were lower than by 2D flow, which is in line with data from both adults and adolescents.^15^, ^16^, ^17^, ^26^ This could be explained by lower spatial or temporal resolution in 4D flow or overestimation of flow volumes by 2D flow as described above. The phantom experiment showed that improving 4D flow spatial resolution from 2.4 mm to 1.5 mm improved accuracy. This study used an acquired temporal resolution of 42 msec, similar to earlier studies and guidelines.^10^, ^30^ Since increased temporal resolution has been shown to improve accuracy of 4D flow volume assessment,^31^, ^32^, ^33^ differences in temporal resolution might also contribute to the difference in flow volumes. Increasing the temporal resolution, in addition to increased spatial resolution, gave better accuracy for volume in the ascending aorta, but the effects in the other vessels were inconclusive, which, as discussed for 2D flow assessment above, may be due to the small diameters of the vessels as well as low flow and thus small differences, in the range of the interobserver variability.

A retrospective comparison of the peak velocity in the isthmus region, for example, the operated area, measured by wave Doppler and the peak velocity measured by 4D flow with 2.4 mm spatial resolution and 42 msec temporal showed no statistically significant difference in mean peak velocity, however a variability that may be of importance for the individual patient. The phantom experiment shows that assessment of peak velocity could likely be improved by increasing both temporal and spatial resolution.

An important limitation of the phantom setup is that it does not include a model of respiratory motion. Previous data in adults have shown that not compensating for respiratory motion may lead to underestimation of flow.^16^ The current study was performed without respiratory gating. Therefore, the respiratory motion may have contributed to underestimation of flow in patients. Improved respiratory motion gating or compensation methods may result in improved accuracy of flow measurements,^34^ although this may come at a scan duration penalty, which is not desirable if keeping the scan time within a feed‐and‐sleep approach.

Internal consistency (i.e., difference between flow volumes in the ascending aorta and the sum of flow volumes in the descending aorta and aortic arch vessels) was better for 4D flow than 2D flow in patients. This may be explained by the simultaneous acquisition of all vessels in the 4D flow data, thus enabling application of the “conservation of mass” principle,^35^, ^36^ compared to the sequential acquisition of flow in vessels for 2D flow, with potential changes in heart rate and other physiological parameters between acquisitions. Furthermore, internal consistency improved with increased spatial resolution in 2D flow and with increased spatial and temporal resolution in 4D flow. The clinical importance of good internal consistency is further discussed below.

### Software Comparison

The 4D flow measurements were performed with two different software packages that used different methods for analysis. Segment transfers the ROI from the 2D flow image plane to the 4D flow data, whereas CAAS MR Solutions uses only 4D flow data. The results suggest that the choice of software does not have large impact on flow measurements. However, it was possible to measure flows in 99% of all vessels with CAAS MR Solutions in contrast to only 75% with the in‐house developed research module for 4D flow to 2D plane reconstruction in Segment.

### Clinical Aspects

Despite 4D flow measuring lower flow volumes than 2D flow, 4D flow may give more precise assessment in neonates, for example for shunts and collateral circulation, in line with results in older children.^26^, ^37^ Acquisition of the 4D flow scan was faster than multiple 2D flow planes when including scan planning time, since 4D flow has a more straightforward preparation and planning. To ascertain that 2D flow planes are correctly positioned and perpendicular to the flow, a good anatomical overview is important. This study used a high‐resolution T1‐weighted black‐blood 3D sequence. Planning of the 4D flow box covering the aorta is significantly simpler than planning multiple 2D flow planes at specific locations, reducing the need for highly skilled MRI technologists. Simplifying and thereby possibly shorten the examination may reduce the need for general anesthesia, which is otherwise often needed in children below the age of 8 years when multiple flow measurements are required. Furthermore, MRI without general anesthesia and contrast agents could prove to be a good substitute for CT with iodinated contrast agents to simultaneously visualize anatomy and measure flow volumes.

Our phantom data suggest that improved temporal resolution can improve measurements of peak velocity. However, the 4D flow sequence used here requires a doubled scan time for a doubling of temporal resolution. From a clinical point of view, the small gain in accuracy does not motivate the cost in scan time. Furthermore, peak velocity can reliably be obtained by Doppler in this patient group. Future developments in 4D flow, for example, using compressed sensing,^38^ machine learning,^39^ or integration with computational fluid dynamics simulations,^40^ may result in 4D flow data being more accurate and precise due to improvements in temporal and spatial resolution without significantly prolonging scan time, making 4D flow an important tool for clinical assessment of neonates with CHD.

There was no statistically significant mean difference between peak velocities in the isthmus region of the aorta measured by 4D flow compared to Doppler echocardiography. Individual variations may be explained by differences in alertness of the neonate. MRI was conducted with the patient sound asleep using feed‐and‐sleep and chloral hydrate as adjunct. In contrast, echo was performed without sedation and thus often on an awake patient, which may have led to higher peak velocities in echo compared to 4D flow MRI. Continuous wave Doppler echocardiography identifies the highest velocity in the course of the ultrasound beam but has low specificity of identifying the precise region of interest, in this study the isthmus region, which may have amplified the disparity in flow velocities measured by echo and MRI.

### Limitations

The study population was small and 4D flow was acquired without respiratory gating. Furthermore, the phantom setup could not simulate respiratory motion, and therefore this effect could not be examined in vitro.

### Conclusion

Assessment of flow in neonates using 4D flow MRI is time efficient and could be acquired with good internal consistency without the use of contrast agents or general anesthesia, thus potentially expanding the use of 4D flow to the youngest and smallest patients.
